# Supplementary material for: Safety and anti-tumour activity of the IgE antibody MOv18 in patients with advanced solid tumours expressing folate receptor-alpha: a phase I trial
Source: Nat Commun. 2023 Jul 25;14:4180. doi: 10.1038/s41467-023-39679-9 (PMC10368744; doi:10.1038/s41467-023-39679-9)
Supplement: Supplementary file 1 — Supplementary Information [file 41467_2023_39679_MOESM1_ESM.pdf]

Supplementary Information

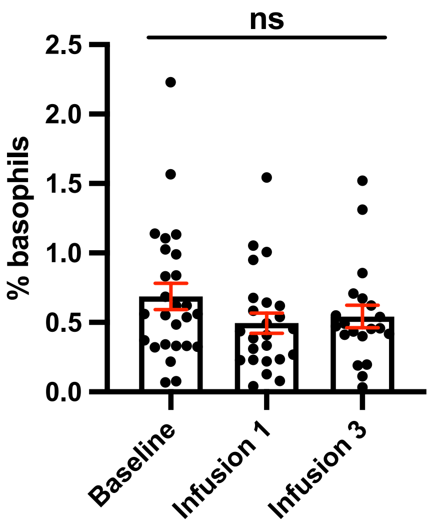

Supplementary Figure 1. Basophils counts. Basophils as a proportion of total circulating leukocytes remained constant over the course of treatment for most patients (at baseline, MOv18 IgE infusion 1, and infusion 3; mean ± SEM; *n* = 26, *n* = 24 and *n* = 20, respectively), unlike the single patient experiencing anaphylaxis (Figure 3f).

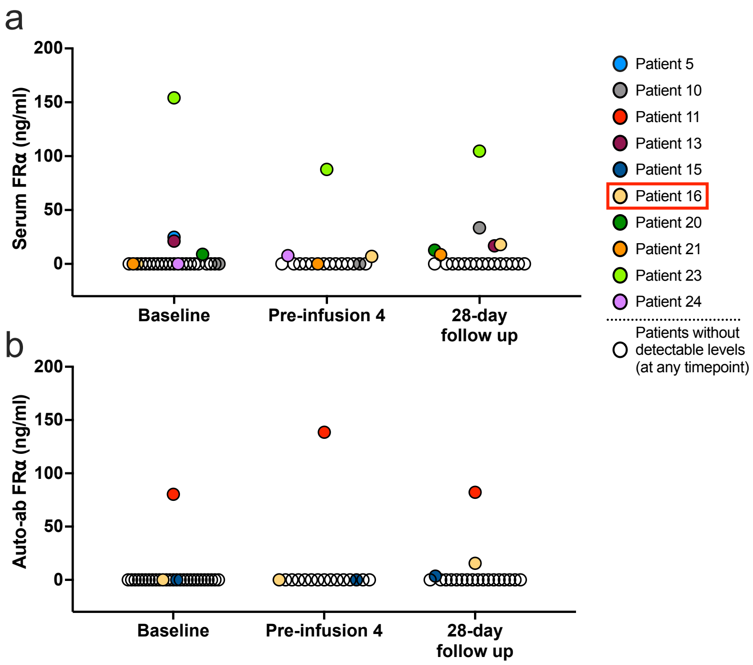

Supplementary Figure 2. Serum FRα and circulating anti-FRα auto-antibody levels. a, serum FRα concentrations measured at baseline, pre-infusion 4 and 28-day follow up (*n* = 26, *n* = 17 and *n* = 21, respectively). **b**, circulating anti-FRα auto-antibody concentrations measured in the serum of patients at baseline, pre-infusion 4, and 28-days follow up (*n* = 26, *n* = 16 and *n* = 20, respectively). Filled points indicate patients who had detectable levels of serum FRα/anti-FRα auto-antibodies at any of the three timepoints. Unfilled points indicate patients who had no detectable levels of either at any of the three timepoints. Only Patient 16 (red box) had detectable levels of both serum FRα and anti-FRα auto-antibodies.

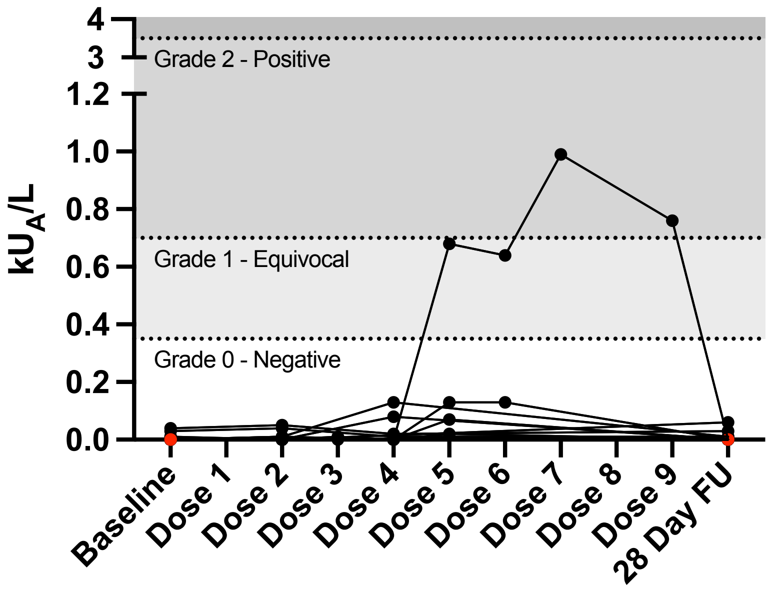

Supplementary Figure 3. Serum anti-alpha-GAL IgE levels. Anti-alpha-GAL IgE antibodies were detected (>0.1 kU_A/L) in three patients following treatment with MOv18 IgE. Seropositivity to alpha-GAL was not detected, before or after treatment, in the patient who experienced anaphylaxis (red points). *n* = 26 patients assessed.

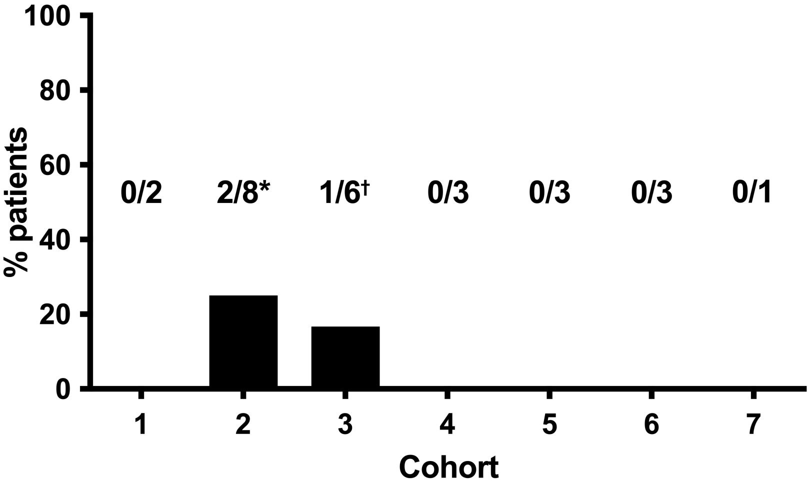

Supplementary Figure 4. Summary of anti-drug antibody (ADA) detection for all patients recruited. At any timepoint and across all dose-level cohorts, ADAs were detected in only 3 patients: in 2 of 8 patients in Cohort 2 (one patient at 28-day follow up, and another at infusion number 6 and at 28-day follow up [*]), and in 1 of 6 patients in Cohort 3 (at 28-day follow up [†]). There was no correlation between ADA titre and toxicity or clinical benefit.

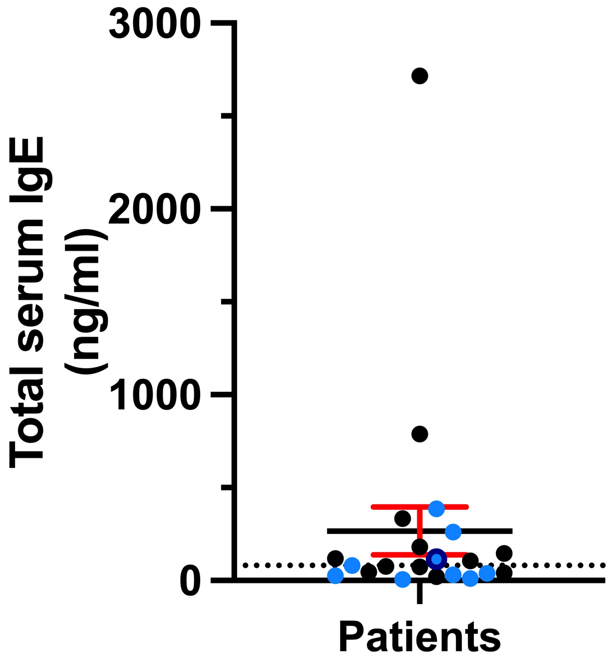

Supplementary Figure 5. Plasma IgE levels. Patients' endogenous IgE levels, where measured, varied widely (mean ± SEM), as is commonly seen in human populations. There was no correlation with observed toxicity or anti-tumour activity. Light blue fill = patients experiencing urticaria; dark blue outline = patient experiencing clinical benefit (n=21).

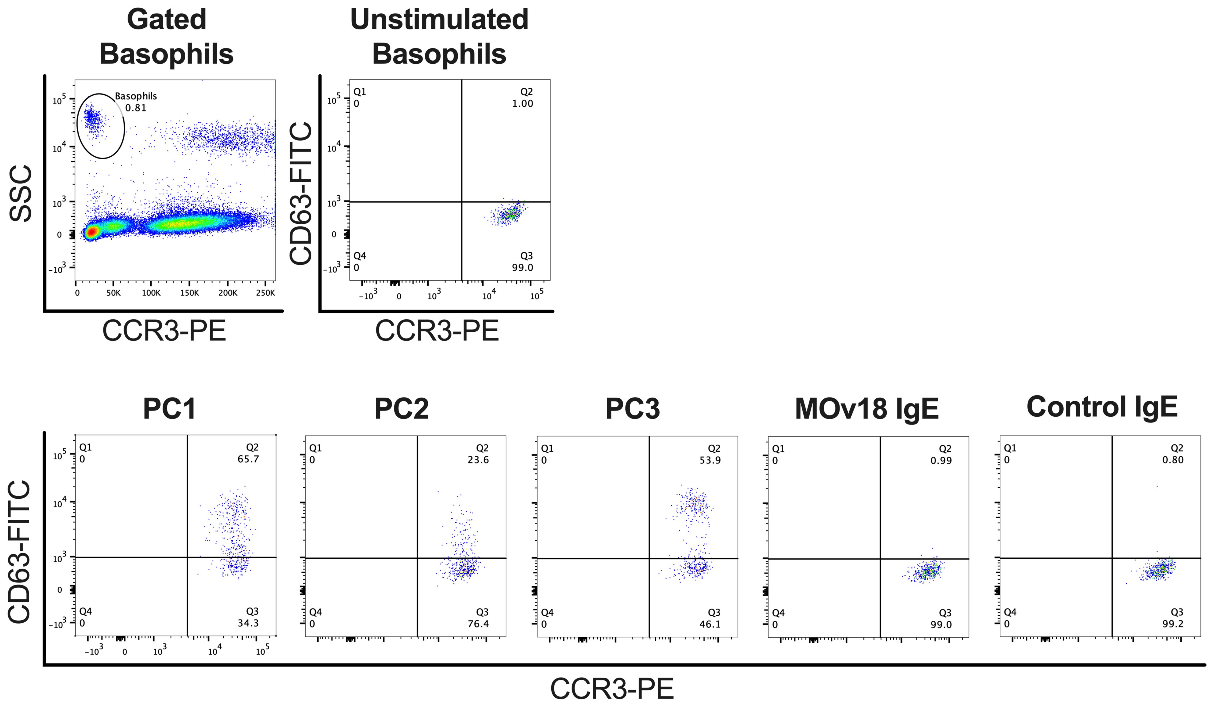

Supplementary Figure 6. Summary of basophil activation test (BAT) flow gating strategy. Gating strategy aimed to identify CCR3^{high}SSC^{low} basophils in unfractionated whole blood samples. Representative plots are shown of patient blood incubated with positive control stimuli (PC1 = anti-FcεRI, PC2 = fMLP, PC3 = anti-IgE), MOv18 IgE, or control IgE.
